# Reorganization of Spinal Cord Microarchitecture by Bioluminescent Optogenetic and Rehabilitative Interventions

**DOI:** 10.3390/cells15060571

**Published:** 2026-03-23

**Authors:** Tatyana Ageeva, Rezeda Shigapova, Aizilya Bilalova, Elizaveta Plotnikova, Amina Akmanova, Albert Rizvanov, Yana Mukhamedshina

**Affiliations:** 1 OpenLab Gene and Cell Technologies, Institute of Fundamental Medicine and Biology, Kazan Federal University, 420008 Kazan, Russia; t.povysheva@gmail.com (T.A.); shigapova.r.r.7@gmail.com (R.S.); rizvanov@gmail.com (A.R.); yana.k-z-n@mail.ru (Y.M.); 2 Department of Histology, Cytology and Embryology, Kazan State Medical University, 420012 Kazan, Russia; 3 Division of Medical and Biological Sciences, Tatarstan Academy of Sciences, 420111 Kazan, Russia

**Keywords:** spinal cord injury, bioluminescent optogenetics, neuronal plasticity, perineuronal nets, dendritic spines, motor rehabilitation

## Abstract

Spinal cord injury (SCI) induces persistent locomotor deficits that are closely associated with maladaptive structural plasticity of spinal neuronal circuits. Although motor rehabilitation improves functional outcomes, the cellular substrates underlying rehabilitation-induced recovery remain incompletely understood, particularly in relation to activity-dependent neuromodulation strategies. Here, we investigated how treadmill-based motor training (TMT) and its combination with bioluminescent optogenetic (BL-OG) stimulation of Hb9 (homebox 9)-positive motoneurons and excitatory interneurons selectively modulate microarchitectural plasticity in the injured rat spinal cord. At the level of gross locomotor assessment, Basso, Beattie and Bresnahan (BBB) scores were comparable between the BL-OG and SCI+TMT groups. Although no statistically significant differences in the total score in rung ladder were observed at 28 days post-injury, animals in the BL-OG group showed a tendency toward a higher ratio of successful hindlimb placements, indicating improved step accuracy. BL-OG stimulation was associated with a slightly greater attenuation of SCI-induced spine abnormalities compared to TMT alone, with significant differences between the experimental groups detected specifically in laminae VIII and IX. These lamina-specific alterations in dendritic integration and dendritic spine composition were accompanied by preservation of wisteria floribunda agglutinin WFA-positive perineuronal net (PNN) architecture. Against this background, reduced glypican-4 (GPC-4) expression and attenuated WFA/GPC-4 colocalization were observed in the SCI+BL-OG group relative to SCI in laminae VII–IX, consistent with activity-dependent modulation of PNN-associated synaptic organization in Hb9-positive neuronal populations. Together, these findings indicate that motor rehabilitation and bioluminescent optogenetic stimulation engage distinct but partially overlapping mechanisms of activity-dependent microarchitectural remodeling, preferentially targeting synaptic and perineuronal net-associated substrates rather than inducing large-scale circuit reorganization. Further studies are warranted to elucidate the mechanisms underlying these distinct plasticity profiles.

## 1. Introduction

Spinal cord injury (SCI) is a complex pathology characterized by severe motor, sensory, and autonomic impairments, leading to prolonged loss of independence and reduced quality of life in patients. The primary mechanical damage triggers a cascade of secondary injury including ischemia, excitotoxicity, oxidative stress, microglial activation, and immune cell infiltration followed by glial scar formation, which creates a chronically unfavorable microenvironment and substantially limits regeneration and functional recovery [1,2]. Against this background, approaches aimed at modulating the post-traumatic microenvironment are being actively developed, including transplantation of stem and progenitor cells, the use of gene therapy, and functional biomaterials [3,4,5]. Nevertheless, decades of research and the diversity of existing approaches have not led to the development of a clinically meaningful and effective therapy for SCI, underscoring the importance of developing integrated, multitarget treatment and rehabilitation strategies.

Among the major therapeutic strategies for SCI, motor rehabilitation occupies a central role. Accumulated clinical evidence demonstrates the effectiveness of various forms of physical therapy, ranging from treadmill-based training to robotic-assisted systems [6,7,8]. Overall, studies indicate that the recovery of stepping movements after SCI is achieved in part through compensatory mechanisms: spared descending pathways, the spinal central pattern generator (CPG) located below the lesion, and sensory feedback jointly contribute to the generation of the rhythmic locomotor pattern [9,10]. Motor rehabilitation enhances this compensatory process by training lumbar networks to respond more effectively to afferent inputs and coordinate motoneuron activity. The structural basis of functional recovery involves pronounced synaptic and axonal plasticity developing in caudal segments under the influence of systematic training. For example, in rats with contusive SCI, treadmill training for 4–8 weeks leads to improvements in locomotor outcomes (BBB scores and paw coordination), indicating neural plasticity and partial restoration of locomotor rhythm generation. At the same time, quantitative morphometric analysis revealed a statistically significant increase in the total dendritic arbor length of alpha motoneurons innervating key hindlimb muscles [11].

The recognition that no single therapeutic intervention can achieve full recovery after severe SCI has driven the active development of comprehensive approaches combining motor rehabilitation with various pharmacological, cellular, and bioengineered strategies. The conceptual basis of such combined strategies lies in simultaneously targeting multiple pathophysiological mechanisms that limit recovery [12,13]. Within such multimodal protocols, motor activity serves as a universal catalyst, enhancing and directing the effectiveness of concomitant interventions by activating endogenous plasticity mechanisms and establishing functionally meaningful patterns of neuronal activity [14].

Bioluminescent optogenetics (BL-OG) is considered a novel and promising method of neuronal stimulation in modern neurobiology [15,16]. As a minimally invasive and highly specific modulation method, BL-OG represents a particularly attractive tool for targeted neuromodulation after SCI. Previously, we developed and validated the genetic construct AAV9-Hb9-LMO3-EYFP for non-invasive BL-OG stimulation and motoneuron recruitment. The AAV9 vector provided efficient CNS neuron transduction, while the Hb9 promoter restricted expression predominantly to motoneurons of lamina IX and ventromedial excitatory interneurons of lamina VIII [17]. Targeting this combined Hb9-positive population allowed us to modulate both the final motor output (motoneurons) and a key excitatory component of the locomotor CPG. This approach is particularly relevant at the L2 segment, which contains critical rhythmogenic circuitry of the lumbar CPG [18]. Following thoracolumbar spinal cord injury, these local Hb9-positive neurons remain anatomically preserved but functionally disconnected from supraspinal input, making them a suitable target for neuromodulatory interventions aimed at restoring locomotor activity. We reasoned that BL-OG stimulation of Hb9-positive neurons, as key elements of the locomotor CPG circuitry, in combination with controlled rehabilitative training, may synergistically facilitate locomotor recovery following SCI.

However, it remains unclear whether such combined interventions induce global remodeling of dendritic architecture or rather lamina- and synapse-specific plasticity, and whether these processes extend to segments distant from the injury epicenter. To address this, we investigated the effects of rehabilitative treadmill training alone and in combination with BL-OG stimulation on the microarchitectural reorganization of the spinal cord following thoracolumbar contusion injury in rats.

## 2. Materials and Methods

### 2.1. Animals

Experiments were conducted using adult female Wistar rats weighing 250–300 g (n = 41). Approval for the animal study was obtained from the local ethics committee of Kazan (Volga region) Federal University (Approval No. 50, dated 26 September 2024). The study was carefully designed to minimize both the number of animals used and the severity of experimental procedures. Rats were maintained under standard housing conditions with a 12 h light/dark cycle and had free access to food and water.

### 2.2. SCI Model and Viral Administration

**Figure 1:**
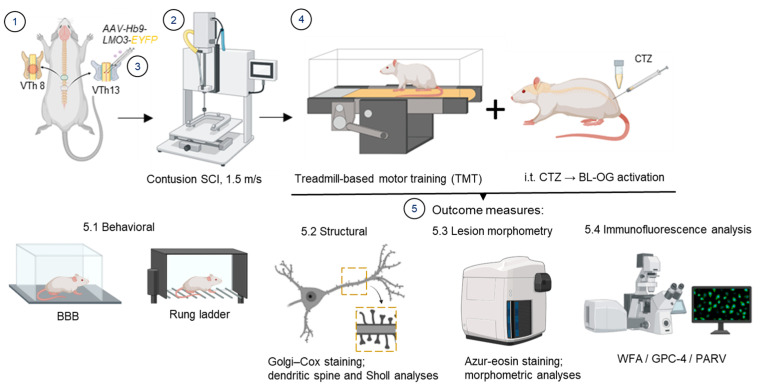
Schematic overview of the experimental design and analytical workflow. (1) Laminectomy was performed at the Th8 and Th13 vertebral levels. (2) A contusive spinal cord injury was induced at the Th8 spinal segment using an impact velocity of 1.5 m/s. (3) Recombinant AAV9 vectors carrying promoter-specific constructs were delivered at the Th13 level to achieve targeted neuronal expression. (4) Beginning at 7 days post-injury, animals underwent daily treadmill-based motor training, either alone or in combination with intrathecal administration of coelenterazine (CTZ) to enable bioluminescent optogenetic stimulation. Outcome measures (5) included: (5.1) behavioral analyses using the BBB locomotor rating scale and the rung ladder walking test; (5.2) structural analyses comprising Golgi–Cox-based dendritic spine analysis and Sholl analysis of dendritic arborization; (5.3) lesion site morphometry using azur-eosin staining to assess spared tissue area; and (5.4) immunofluorescence analysis of perineuronal nets (PNNs). Created in BioRender. Mayasin, Y. (2026) https://BioRender.com/2ij6mom.

Rats were operated on under general anesthesia using Zoletil (20 mg/kg, intramuscularly, Carros, Virbac) following premedication with xylazine hydrochloride (10 mg/kg, Nita-Pharm, Saratov, Russia). A laminectomy was performed at the vTh8 (vertebrate thoracic segment 8) level, after which a contusion model of traumatic SCI was induced using an Impact One Stereotaxic Impactor (Leica Microsystems GmbH, Wetzlar, Germany) (Figure 1). The impact velocity was 1.5 m/s, corresponding to a mild degree of injury.

Animals underwent intraspinal injections at the L1–L2 level using a syringe pump (KD Scientific, Holliston, MA, USA) at a rate of 0.7 μL/min immediately after SCI. Injections were performed at coordinates 0.5–1 mm lateral to the midline and 1.5 mm in the ventral direction. Animals received AAV9-Hb9-LMO3-EYFP at a dose of 3.5 × 10^13^ genome copies/mL in a total volume of 5 μL of sterile 0.9% NaCl (Supplementary S1). The design and validation of the viral constructs were described in our previous study [17]. To provide access to the spinal cord, a Th13 laminectomy was performed prior to injection.

The wound was then closed in layers, and all experimental animals received intramuscular enrofloxacin (10 mg/kg, Novakorm, Ekaterinburg, Russia) once daily for 7 days postoperatively. During the postoperative period, due to the development of urinary dysfunction, manual bladder expression was performed twice daily until spontaneous bladder function was restored.

The rats were randomly assigned to 4 groups: group 1 (Sham, n = 8): laminectomy at vTh8 and vTh13 without SCI or viral transduction; group 2 (SCI, n = 10): contusion SCI at vTh8; group 3 (SCI+TMT, n = 10): contusion SCI at vTh8, followed by treadmill-based motor training (see Section 2.3); group 4 (BL-OG, n = 10): contusion SCI at vTh8, laminectomy at vTh13, intraspinal AAV9-Hb9-LMO3-EYFP injection at L1–L2, treadmill-based motor training (see Section 2.3), and intrathecal CTZ administration (see Section 2.4). Intact rats (n = 3), matched for age and sex, were included as reference controls for dendritic spine and Sholl analyses. The experimental design is summarized in Table 1.

### 2.3. Treadmill Training

Physical exercise on the treadmill was performed using an IITC Life Science Treadmill for Mice and Rats (800 Series Treadmill). Rehabilitation was initiated on day 7 after SCI to capture the early window of activity-dependent plasticity following resolution of spinal shock, and was carried out 5 days per week. Training sessions lasted 20 min and were conducted twice daily with an interval of at least 2 h. Rats were trained at speeds ranging from 6 cm/s (3.6 m/min) to 21 cm/s (12.6 m/min), depending on their functional status, with a gradual increase in workload. The rehabilitation program lasted 3 weeks. In the absence of plantar paw placement, forced plantar placement of the hind limbs was performed during treadmill training according to the method described by Hayashibe et al. [19].

### 2.4. Bioluminescent Optogenetic Stimulation

Bioluminescent activation of LMO3-expressing neurons was achieved by intrathecal administration of coelenterazine (NanoLight Technologies, Pinetop, AZ, USA, Cat. No. 3013). One week after intraspinal AAV9-Hb9-LMO3-EYFP delivery, rats received daily intrathecal CTZ injections (five per week) during weeks 2–4.

For each injection, CTZ was freshly reconstituted by dissolving the lyophilized reagent in sterile, degassed water for injection. The water was degassed immediately before use by brief ultrasonic treatment to eliminate dissolved gases. The final CTZ solution was protected from light and administered promptly after preparation. Intrathecal injections were performed under isoflurane anesthesia (4%) in a 1:1 mixture of oxygen and air. Rats were positioned in sternal recumbency with lumbosacral flexion, and a 25G Hamilton syringe was inserted at the L6–S1 interspace. Successful access to the intrathecal space was confirmed by cerebrospinal fluid appearance or a transient tail flick. A single 20 µL bolus of freshly prepared CTZ (150 µg per injection) solution was delivered at each session.

### 2.5. BBB Locomotor Rating Scale

Voluntary locomotor activity was evaluated using the BBB locomotor rating scale by two blinded observers who were unaware of the animals’ affiliation. Animals were habituated to the open-field arena for 3 days pre-surgery. Beginning on day 7 after SCI, rats were assessed twice weekly at the same time of day by placing them in the center of the open field and scoring locomotor parameters. The final BBB scores were calculated as the average of the two independent evaluations. On days when behavioral testing coincided with training and CTZ administration, assessments were performed at least 1 h prior to treadmill training and intrathecal injection.

### 2.6. Rung Ladder Test

To habituate animals to the experimental conditions, rats were pre-trained on the rung ladder apparatus (OpenScience, Krasnogorsk, Russia) for 3 days prior to surgery. Behavioral testing was initiated at 7 days post-injury (dpi) and conducted twice weekly until the end of the experiment (28 dpi). Video recordings were acquired using a Canon EOS 250D camera equipped with an EF-S 18–55 mm f/4–5.6 IS STM lens mounted in a fixed position. For each animal, a 3 min video recording was obtained, during which rats performed an average of four complete traverses in alternating directions (left to right and right to left). This protocol enabled assessment of motor performance of all four limbs. Limb placement was evaluated based on paw positioning on the rungs and the occurrence of limb slippage between rungs in the event of a misstep. During video analysis, a step was scored if movement in three joints was observed (corresponding to ≥4 points on the BBB scale according to Metz et al. [20], as well as in cases of full plantar paw placement (≥9 points). This modification of the standard rung ladder protocol allows for a more sensitive assessment of locomotor function in injured animals by accounting for all limb movements, including those not yet achieving consistent plantar support. Quantitative analysis was performed across four traverses per animal and included the following parameters: (1) total number of steps; (2) number of errors [21]; and (3) traversal score for each pass [20].

### 2.7. Tissue Sparing Analysis

A 0.2 cm segment of the spinal cord centered on the lesion epicenter at the Th8 vertebral level (defined as point 0; range: 0.1 cm rostral to 0.1 cm caudal) was dissected and fixed in 10% neutral buffered formalin for 24 h. Specimens were then processed through a graded series of isopropyl alcohol, isopropyl alcohol–mineral oil mixture, mineral oil, and paraffin. Tissue blocks were embedded in paraffin wax. For each specimen, 10 slides were prepared, each containing 10 serial transverse sections cut at 5 µm thickness. Sections were stained with azur-eosin and digitized using an Aperio ScanScope whole-slide imaging system (Leica Biosystems, Nussloch, Germany) at ×20 magnification. For each experimental group, the total area of pathological cavities and the area of spared tissue were measured on transverse spinal cord sections. The cavity area was calculated by summing all post-traumatic cysts with an area ≥ 800 µm^2^ using Aperio ImageScope software (v12.4.6; Leica Biosystems, Wetzlar, Germany).

### 2.8. Dendritic Spine Analysis

At 28 days after the laminectomy or SCI rats were anesthetized and transcardially perfused with phosphate-buffered saline (PBS, pH 7.4, 4 °C, PanEco, Moscow, Russia) followed by 10% neutral buffered formalin (Biovitrum, St. Petersburg, Russia). Spinal cord samples from the Th9 and L2 segments were impregnated with Golgi–Cox solution at room temperature in the dark for 14 days. After impregnation, the tissue was cryoprotected in 30% sucrose at 4 °C for 24 h to reduce sectioning artifacts. Transverse sections (100 µm thick) were cut using a cryostat and collected in 12-well plates containing phosphate-buffered saline. Sections were then developed in D-76 developer for 5 min and rinsed in distilled water. Fixation was performed in a solution of ammonium thiocyanate and sodium thiosulfate for 15 min, followed by washing in water. For final preparation, sections were transferred to phosphate buffer, dehydrated in 96% ethanol, cleared in xylene, and covers lipped with Vitrogel (Biovitrum, St. Petersburg, Russia). Golgi-stained transverse sections were examined using a PrimoStar 3 microscope (Carl Zeiss, Oberkochen, Germany). Neurons were selected according to the following criteria: (1) localization within laminae VII–X; (2) complete and uniform Golgi impregnation without signs of incomplete staining; and (3) fully visible somata and dendritic arbors unobscured by nonspecific precipitates, blood vessels, glial elements, or overlapping dendrites of adjacent impregnated neurons. For analysis, 3–6 neurons per lamina were examined per section (n = 5 sections) from animals (n = 3 per group).

The proportional density of thin, mushroom, stubby, and wide spines was assessed according to previously established criteria [22,23]. Thin spines were defined by a neck diameter smaller than spine length and a head diameter larger than the neck diameter. Mushroom spines were characterized by a neck shorter than the longitudinal head dimension and a larger head diameter compared with thin spines. Stubby spines lacked a distinguishable neck and head, with a diameter greater than or equal to their length. Wide spines, morphologically similar to stubby spines, were defined by a total spine length exceeding the spine diameter.

### 2.9. Sholl Analysis

For Sholl analysis, 3–6 impregnated isolated neurons located in lamina IX were imaged for each animal (n = 3). Using a digital drawing tool, images of these neurons were reconstructed to visualize dendritic arborization. Using ImageJ (v.1.54p; National Institutes of Health, Bethesda, MD, USA), the image threshold was adjusted, and the Sholl analysis plugin was applied with concentric shells starting at 10 µm from the soma center, with a step size of 10 µm, extending to the tip of the longest dendrite. The number of dendritic intersections with each concentric shell was recorded as a measure of dendritic branching complexity. For between-group comparison, values from all analyzed neurons were averaged per animal.

### 2.10. Immunofluorescent Staining and Quantitative Analysis of Perineuronal Nets (PNNs)

For immunofluorescent analysis, a fragment of the spinal cord (L2) was collected and additionally fixed in 10% buffered formalin overnight, followed by incubation in 30% sucrose solution (Dia-M, Moscow, Russia). Samples were placed in a tissue-freezing medium Tissue-Tek O.C.T. Compound (Sakura Finetek, Tokyo, Japan), and then 20 μm thick cross-sections of the spinal cord were obtained using a cryostat FS800A (RWD Life Science, Shenzhen, China).

For antigen identification, the sections were incubated overnight at 4 °C with primary antibodies against GPC4 (1:100, Cloud-Clone Corp., Wuhan, China), parvalbumin (PARV) (1:400, Sigma-Aldrich, St. Louis, MO, USA), and wisteria floribunda agglutinin (WFA) (Vector Laboratories, 1:500, Newark, CA, USA). On the following day, the sections were washed in PBS and incubated with secondary antibodies for 2 h at room temperature. To visualize cell nuclei, the sections were additionally stained with 4′,6-diamidino-2-phenylindole (DAPI, Sigma-Aldrich, USA), thoroughly washed in PBS, and mounted in ImminoHistomount (Santa Cruz Biotechnology, sc-45086, Dallas, TX, USA).

Immunofluorescence imaging was performed using an LSM 700 confocal microscope (Carl Zeiss AG, Oberkochen, Germany). All images were acquired using identical confocal microscope settings (laser intensity, gain, and offset). Analysis was performed on digitized images (0.5 × 0.5 mm; 1080 × 1080 pixels) in Laminae VII, VIII, IX, and X of the gray matter in each of 6 spinal cord sections (n = 5 per group) using ImageJ software (v.1.54p; National Institutes of Health, Bethesda, MD, USA). The following measurements were performed: (1) the number of PNN vertices, (2) area and (3) perimeter of perineuronal net (PNN) vertices, and integrated density of WFA, glypican-4 (GPC-4), and PARV. For the analysis, each PNN surrounding a neuronal soma was manually outlined as a polygon, and the area, perimeter, and number of vertices were then measured [24].

### 2.11. Statistical Analysis

All statistical analyses were performed using Origin 10.0 SR0 (OriginLab Corporation, Northampton, MA, USA) and GraphPad Prism 8.0.1 (GraphPad Software, San Diego, CA, USA). Data obtained from the BBB rating scale, rung ladder walking test, dendritic spine, sholl, and colocalization coefficient analyses were assessed using the non-parametric Kruskal–Wallis test followed by Dunn’s multiple comparisons post hoc test due to non-normal data distribution. All remaining quantitative data were analyzed using one-way analysis of variance (one-way ANOVA) followed by Tukey’s post hoc test. Statistical significance was defined as *p* < 0.05, *p* < 0.01, and *p* < 0.001.

## 3. Results

### 3.1. Hindlimb Locomotion and Tissue Sparing

**Figure 2:**
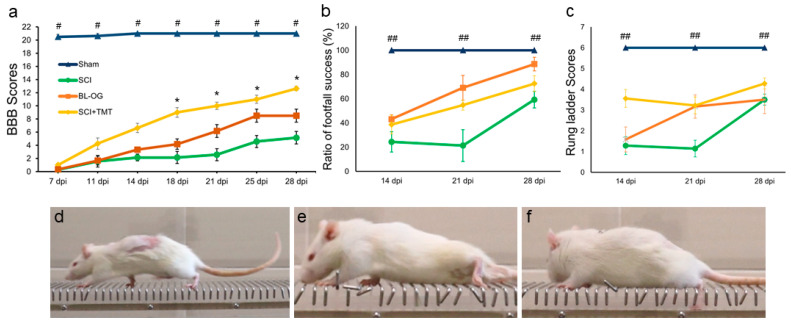
(**a**) Results of the BBB rating scores (Y axis) in the experimental groups at 7–28 dpi or laminectomy (X axis). ^#^ *p* ≤ 0.01 compared to SCI, SCI+TMT and BL-OG. * *p* ≤ 0.05 compared to SCI. Kruskal–Wallis test with Dunn’s post hoc test. Results of the analysis of the (**b**) percentage of correct steps and (**c**) the rung ladder scores (Y axis) in the experimental groups at 14–28 dpi or laminectomy (X axis). ^##^ *p* ≤ 0.05 compared to SCI, SCI+TMT and BL-OG. Kruskal–Wallis test with Dunn’s post hoc test. Photographs illustrating three categories of hind leg placement on the bar: (**d**) plantar foot placement, (**e**) partial foot placement, and (**f**) complete miss.

Assessment of locomotor function using the BBB scale revealed a progressive improvement in motor performance in the BL-OG and SCI+TMT groups (Figure 2a, Supplementary Table S1). During the first post-injury week, differences between the experimental groups were minimal, and all animals exhibited persistent hindlimb paralysis. Starting from 11 dpi in the SCI+TMT group and from 18 dpi in the BL-OG group, a progressive increase in locomotor activity was observed compared with the SCI control group.

From 18 dpi onward, the highest BBB scores were recorded in the SCI+TMT group, with a peak of motor performance at 28 dpi. Statistically significant differences were detected when compared with the SCI group (*p* ≤ 0.05) [18 dpi: SCI+TMT 9.0 ± 0.73 vs. SCI 2.14 ± 0.79; 28 dpi: SCI+TMT 12.63 ± 0.32 vs. SCI 5.14 ± 0.42]. The BL-OG group reached its maximum locomotor score at 25 dpi (8.5 ± 0.99), which was 86% higher than that of the SCI group (4.57 ± 0.57), although this difference did not reach statistical significance. No statistically significant differences in BBB scores were observed between the BL-OG and SCI+TMT groups throughout the entire observation period. Animals in the Sham group exhibited consistently maximal BBB scores across the experiment and differed significantly from all other experimental groups.

Locomotor performance assessed using the rung ladder walking test was initiated at 14 dpi, taking into account the severity of neurological impairment. Between 14 and 28 dpi, the BL-OG group demonstrated a higher proportion of correct steps, reaching a maximum of approximately 88.6% at 28 dpi, compared with the SCI+TMT (~72.4%) and SCI (~59.3%) groups; however, these differences were not statistically significant (Figure 2b,d–f). The Sham group consistently exhibited maximal performance throughout the experimental period and differed significantly from all experimental groups (*p* ≤ 0.05).

Throughout the observation period, the SCI+TMT group exhibited both minor decreases and marked increases in rung ladder scores, without statistically significant differences compared with the BL-OG and SCI groups (Figure 2c). At 28 dpi, the highest mean scores among the experimental groups were recorded [SCI+TMT: 4.27 ± 0.26; BL-OG: 3.50 ± 0.67; SCI: 3.50 ± 0.66]. Animals in the Sham group consistently demonstrated maximal scores (6 points) and differed significantly from all experimental groups (*p* ≤ 0.05).

To assess whether the observed functional changes were accompanied by differences in tissue preservation, tissue sparing analysis was performed at the lesion epicenter. The percentage of spared tissue did not differ significantly among the SCI (85.17%), SCI+TMT (86.87%), and BL-OG (84.08%) groups (Kruskal–Wallis, H = 0.34, *p* = 0.84; Supplementary Figure S1).

### 3.2. Morphological Characteristics of Motoneuron Dendritic Arborization

**Figure 3:**
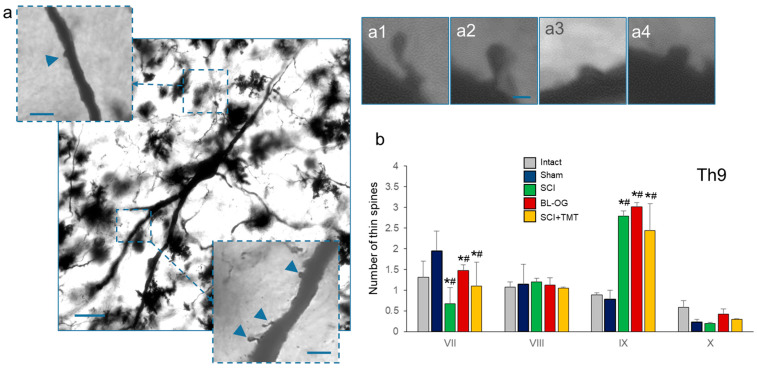
(**a**) Motoneuron, dendritic segment, and dendritic spines. Motoneurons selected for dendritic spine quantification from the lumbar (L2) spinal cord segment. Scale bar: 200 µm. Arrows without dots indicate dendritic spines within the dendritic segment. Scale bar: 30 µm. (**a1**–**a4**) Representative types of dendritic spines: (**a1**) thin, (**a2**) mushroom, (**a3**) stubby, and (**a4**) wide. Scale bar: 3 µm. (**b**) Distribution of thin dendritic spines across spinal cord lamina in the Th9 segment. Statistical analysis was performed using the Kruskal–Wallis test followed by Dunn’s multiple comparisons post hoc test; *p* < 0.05: *—comparison with the Intact, and ^#^—with the Sham group.

Morphological and immunohistochemical analyses were performed at the Th9 segment (peri-lesional level, caudal to the injury site) and the L2 segment (site of AAV9 transduction and lumbar CPG circuitry) to assess the spatial extent of injury- and treatment-related plasticity. All somato-dendritic morphological parameters subjected to quantitative analysis are presented as mean values for each experimental group in Table 2. The morphological characteristics of the analyzed neurons were consistent with medium-sized motoneurons located in lamina IX of the spinal cord at the Th9 and L2 levels (Figure 3(a,a1–a4)). In both spinal cord segments, the density of thin, mushroom, and stubby dendritic spines in the SCI group was increased approximately 2–3-fold compared with the Intact and Sham groups. In the SCI+TMT and BL-OG groups, the densities of these spine types were reduced relative to the SCI group and exhibited intermediate values, remaining higher than those observed in the control groups. The density of wide dendritic spines did not differ significantly between the experimental groups. Significant differences were detected between the Intact and Sham groups and between the Sham and SCI groups (*p* < 0.05).

**Figure 4:**
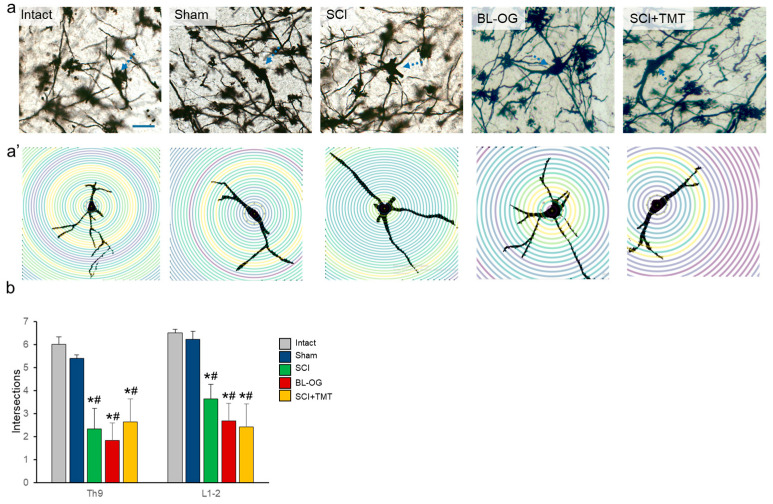
(**a**) Lamina IX of the lumbar spinal cord at the L2 segment, Golgi staining. Arrows indicate motoneurons selected for dendritic intersection analysis. Scale bar: 200 µm. (**a′**) Lower panel: dendritic intersections measured using Sholl analysis in the Intact, Sham, SCI, BL-OG and SCI+TMT groups. Representative isolated motoneurons selected for reconstruction are shown. Individual Sholl profiles of the representative neurons shown in (**a′**) are provided in Supplementary Figure S2. (**b**) Quantitative assessment of dendritic intersections using Sholl analysis. Statistical analysis was performed using the Kruskal–Wallis test followed by Dunn’s multiple comparisons post hoc test; *p* < 0.05: *—comparison with the Intact, and ^#^—with the Sham group.

Parameters dependent exclusively on total dendritic length and branching pattern exhibited minimal variation across experimental groups. On average, motoneurons displayed comparable numbers of primary dendrites, as well as similar numbers of dendritic segments and segments per dendrite (Figure 4a,a′), with no statistically significant differences between groups.

In contrast, quantitative Sholl analysis revealed pronounced group-dependent differences in dendritic network organization (Figure 4b). In the Intact group, the highest numbers of dendritic intersections were observed at both the Th9 (6.00 ± 0.33) and L1–L2 (6.50 ± 0.17) levels. Slightly lower values were detected in the Sham group (Th9: 5.39 ± 0.35; L1–L2: 6.22 ± 0.35), although no statistically significant differences were found compared with the Intact group. Following SCI group exhibited a marked reduction in the number of dendritic intersections at both analyzed levels, reaching 2.33 ± 0.89 at Th9 and 3.64 ± 0.63 at L2. These values were significantly lower than those observed in the Intact and Sham groups (*p* < 0.05).

Representative Sholl profiles of lamina IX motoneurons at the L2 level (Supplementary Figure S2) illustrate the spatial distribution of dendritic intersections as a function of distance from the soma. In the Intact and Sham groups, dendritic intersections were maintained across a broad range of radii (up to 350–400 µm), with peak values observed at proximal distances (40–80 µm). Following SCI, the overall extent of the dendritic arbor appeared preserved; however, intersection counts showed greater variability across radii. The SCI profile displayed lower peak intersection counts (~3 intersections) compared with Intact and Sham (~5 intersections), consistent with reduced dendritic branching complexity observed at the group level. In the BL-OG and SCI+TMT groups, dendritic arbors extended up to 240–300 µm, with peak intersections concentrated at proximal distances.

Within-group analysis of the SCI group revealed a significant segment-dependent effect: the number of dendritic intersections at L2 was significantly higher than at Th9 (*p* < 0.01), indicating a more pronounced reduction in dendritic arborization at the thoracic level. In the experimental treatment groups SCI+TMT and BL-OG, the mean numbers of dendritic intersections were 2.63 ± 0.75 and 1.84 ± 0.39 at Th9, and 2.43 ± 0.75 and 2.69 ± 0.99 at L1–L2, respectively. No statistically significant differences were detected between these groups and the SCI group at the corresponding spinal levels.

### 3.3. Perineuronal Net Organization

**Figure 5:**
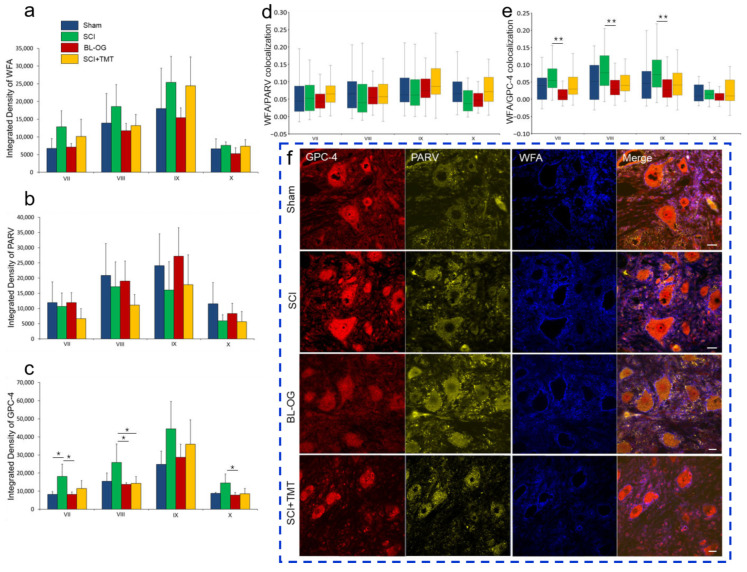
Integrated density of (**a**) WFA, (**b**) PARV, (**c**) GPC-4 and colocalization coefficient of (**d**) WFA/PARV and (**e**) WFA/GPC-4 (Y axis) in Lamina VII-X (X axis) in the experimental groups at 28 dpi or laminectomy. * *p* < 0.05, one-way ANOVA with Tukey’s post hoc test. ** *p* < 0.01, Kruskal–Wallis test with Dunn’s post hoc test. (**f**) Confocal microscopy of the spinal cord at the level of L1-2 in the Lamina IX in experimental groups at 28 dpi or laminectomy: GPC-4 (red), PARV (yellow), WFA (blue). Scale: 20 µm.

Using immunofluorescence (IF), we analyzed WFA, GPC-4, and PARV expression in laminae VII–X of the lumbar (L1–L2) spinal cord gray matter across experimental groups. The integrated density of WFA was highest in lamina IX (L1–L2); however, no significant between-group differences were detected [IX: Sham 17,996 ± 11,396; SCI 25,399 ± 7356; SCI+TMT 24,435 ± 8153; BL-OG 15,415 ± 2754] (Figure 5a). The lowest WFA integrated density values were observed in laminae VII and X, likewise without significant between-group differences. PARV integrated density in the analyzed laminae of the L1–L2 segments showed a pattern similar to that of WFA, with no significant between-group differences detected; the highest and lowest PARV expression levels were observed in lamina IX and lamina X, respectively (Figure 5b).

In contrast, GPC-4 integrated density in lamina VII was increased in the SCI group compared with Sham (*p* ≤ 0.05) and BL-OG (*p* ≤ 0.01) [VII: SCI 18,143 ± 6760 vs. Sham 8229 ± 1521 and BL-OG 8178 ± 1419]. In lamina VIII, GPC-4 integrated density was also higher in the SCI group compared with BL-OG and SCI+TMT [VIII: SCI 25,867 ± 10,196 vs. BL-OG 13,719 ± 1010 and SCI+TMT 14,337 ± 3689, *p* ≤ 0.05]. A similar pattern was observed in lamina X, where GPC-4 integrated density was higher in SCI compared with BL-OG [X: SCI 14,491 ± 4966 vs. BL-OG 7872 ± 1420, *p* ≤ 0.05]. The highest GPC-4 integrated density was detected in lamina IX (L1–L2), although no significant between-group differences were observed (Figure 5c).

To assess the relationship between PNNs and neuronal functional specialization, we additionally quantified the colocalization coefficients for WFA/PARV and WFA/GPC-4 in laminae VII–X of the L1–L2 gray matter. No significant between-group differences were detected for the WFA/PARV colocalization coefficient, and within-group differences were not observed, except for laminae VII and IX in the BL-OG group (Figure 5d). In contrast, the WFA/GPC-4 colocalization coefficient was higher in the SCI group than in the BL-OG group in laminae VII, VIII, and IX [VII: SCI 0.067 ± 0.059 vs. BL-OG 0.017 ± 0.029, *p* ≤ 0.001; VIII: SCI 0.084 ± 0.064 vs. BL-OG 0.033 ± 0.031, *p* ≤ 0.01; IX: SCI 0.078 ± 0.056 vs. BL-OG 0.033 ± 0.036, *p* ≤ 0.01] (Figure 5e,f).

**Figure 6:**
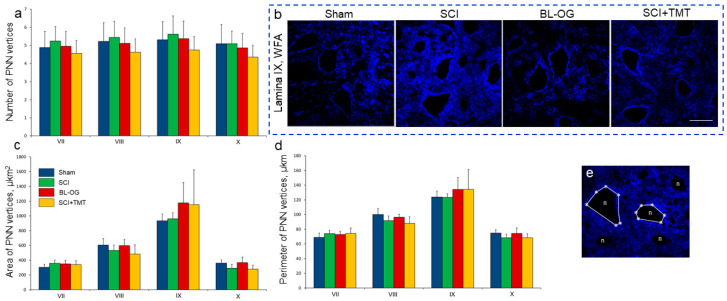
Quantitative analysis of PNN microstructure: (**a**) number of PNN vertices, (**c**) the area and (**d**) the perimeter of PNN vertices (Y axis) in the experimental groups at 28 dpi or laminectomy. No significant intergroup differences were found, one-way ANOVA with Tukey’s post hoc test. (**b**) Confocal microscopy of the spinal cord at the L1-2 level in lamina IX at 28 dpi or laminectomy. Scale: 50 µm. (**e**) Example of manual PNN vertices selection. n—neuron. *—selected PNN vertices. Lines are the outlined perimeter of PNN.

Across all groups (Sham, SCI, SCI+TMT, and BL-OG), no significant differences were detected in the number of PNN vertices of the analyzed neurons in laminae VII–X at the Th9 and L2 levels, which ranged from 4 to 7 (Figure 6a,b,e). The PNN area and perimeter varied significantly depending on neuronal location, with the largest values observed in lamina IX; however, no significant between-group differences were detected (Figure 6c,d).

## 4. Discussion

We found that motor rehabilitation and its combination with selective neuronal stimulation were associated with improved locomotor performance after SCI. At the level of gross locomotor assessment, BBB scores were comparable between the BL-OG and SCI+TMT groups, although scores had not yet plateaued by 28 dpi, reflecting the early chronic time point chosen for analysis. The rung ladder test revealed functional differences that were not captured by the BBB integral assessment of gross locomotion. Although no statistically significant differences in the total score were observed at 28 days post-injury, animals in the BL-OG group showed a tendency toward a higher ratio of successful hindlimb placements, indicating improved step accuracy. These changes point to modulation of coordination-related components of locomotion rather than basic motor output. The observed pattern is consistent with reports that Hb9 expression is not restricted to motoneurons but extends to a population of ventromedial excitatory interneurons implicated in the organization of locomotor rhythm and coordination [25,26]. The observed improvement in the BL-OG group is consistent with results reported by Petersen et al. [27], who demonstrated that BL-OG stimulation of spinal neurons accelerates locomotor recovery after SCI, with no effect attributable to CTZ, bioluminescence, or their breakdown products alone. Although the present study did not include an analogous CTZ-only control, the documented specificity of the BL-OG response [27,28], together with the known antioxidant properties of CTZ [29], suggests that substrate-mediated non-specific effects are unlikely but cannot be entirely excluded.

Functionally selective behavioral effects prompted a lamina-specific analysis of the morphological organization of spinal neurons. SCI in the present study caused a marked reduction in dendritic intersections of lamina IX motoneurons, as revealed by Sholl analysis, while total dendritic length and primary branching remained largely preserved. This dissociation indicates impaired dendritic integration rather than overt dendritic degeneration and parallels observations across multiple CNS pathologies in which functional disconnection precedes structural loss. Reduced Sholl complexity despite preserved dendritic length has been reported in peri-infarct cortical neurons after ischemia [30], hippocampal neurons following repetitive mild traumatic brain injury [31], and early stages of Alzheimer’s disease, where dendritic simplification reflects disrupted synaptic integration preceding neuronal loss [32]. Accordingly, the reduction in dendritic intersections observed here likely represents a conserved response to injury characterized by impaired integrative capacity.

SCI-induced alterations in dendritic integration were segment-dependent, with thoracic motoneurons exhibiting a greater reduction in Sholl intersections than lumbar motoneurons. This rostrocaudal gradient is consistent with evidence that neurons located closer to the lesion epicenter sustain more severe impairments, whereas caudal neurons retain greater plastic potential due to partial preservation of descending and propriospinal inputs [33]. Concomitantly, analysis of dendritic spine composition across laminae VII–X revealed pronounced SCI-induced remodeling, including increased densities of thin and mushroom-shaped spines. Similar spine profiles have been reported after SCI and interpreted as maladaptive stabilization of excitatory synapses rather than compensatory synaptogenesis [34,35]. Comparable combinations of reduced dendritic integration and unstable spine remodeling have also been described after ischemia and in early Alzheimer’s pathology, supporting the interpretation that these changes reflect pathological synaptic reorganization [30,32].

Treadmill-based motor training did not significantly restore dendritic integration but partially normalized dendritic spine composition. This dissociation aligns with evidence that rehabilitation after SCI primarily modulates synaptic efficacy and local connectivity rather than reconstructing dendritic architecture [33,36]. Similar effects have been reported in ischemia and traumatic brain injury models, where rehabilitative or environmental interventions altered spine morphology without robust changes in dendritic complexity [26,27], consistent with the view that dendritic spines represent a more sensitive substrate for activity-dependent plasticity than dendritic arbors [37].

Bioluminescent optogenetic stimulation resulted in a slightly greater attenuation of SCI-induced spine abnormalities compared to motor training alone, with significant differences between the BL-OG and SCI+TMT groups detected specifically in laminae VIII and IX. These effects remained confined to lamina-specific synaptic remodeling and were not accompanied by restoration of dendritic integration, consistent with prior demonstrations that cell-type-specific activation biases synaptic remodeling and intrinsic excitability without inducing large-scale dendritic or circuit reorganization [38,39]. These results are consistent with prior BL-OG studies demonstrating neuroplasticity-related effects: upregulation of synaptic remodeling markers (GAP-43, PSD-95, MAP2) after spinal cord contusion [27,40] and enhanced axon regeneration after peripheral nerve injury [15]. Traditional optogenetic stimulation after ischemic stroke similarly promoted GAP-43 upregulation and axonal sprouting with functional recovery [41,42]. However, as the SCI+TMT group did not receive vehicle intrathecal injections or repeated isoflurane anesthesia, non-specific effects of the injection procedure on the observed BL-OG group outcomes cannot be fully excluded. Accordingly, the observed differences between the SCI+BL-OG and SCI+TMT groups should be considered exploratory.

After SCI, structural alterations in neurons predominantly affect synaptic organization and dendritic integration, whereas the architecture of perineuronal nets may remain preserved, particularly in segments located caudal to the lesion epicenter [30,43,44]. In the present study, lamina-specific alterations in dendritic integration and dendritic spine composition were accompanied by preservation of WFA-positive PNN architecture in laminae VII–X. Against this background, reduced GPC-4 expression and attenuated WFA/GPC-4 colocalization in laminae VII–IX were observed in the SCI+BL-OG group relative to SCI, consistent with normalization of injury-induced changes in Hb9-positive neurons. Previous studies have shown that changes in neuronal activity, both in vivo and in vitro, are accompanied by activity-dependent induction of perineuronal net components, including proteoglycans, without disruption of overall PNN structural integrity [45,46]. Lamina-specific upregulation of GPC-4 in lumbar motoneurons following thoracic SCI has also been previously reported and interpreted as part of post-injury synaptic reorganization [47].

In addition to PNN-associated remodeling, the synaptic microenvironment of lamina IX motoneurons includes parvalbumin-immunoreactive elements—primarily proprioceptive Ia afferent terminals originating from dorsal root ganglia neurons, which form dense monosynaptic contacts on α-motoneurons [48,49,50,51]. PARV-positive V1-derived interneurons, including Ia inhibitory interneurons, may also contribute to this signal [50,52]. Despite the known vulnerability of proprioceptive Ia afferent synapses to spinal cord injury [53], PARV integrated density in lamina IX did not differ significantly between groups in our study. This finding is consistent with evidence that parvalbumin-immunoreactive terminals on motoneurons may increase after complete spinal transection, potentially reflecting compensatory sprouting of spared afferents [54] or neuroprotective upregulation of calcium-buffering capacity [55]. The preserved PARV signal thus likely reflects a balance between injury-induced synaptic terminal loss and compensatory mechanisms. Accordingly, the WFA/PARV colocalization coefficient also remained unchanged, suggesting that the spatial relationship between PARV-positive elements and perineuronal nets in lamina IX is maintained at this time point regardless of treatment condition.

## 5. Conclusions

Motor rehabilitation and bioluminescent optogenetic stimulation of Hb9-positive motoneurons and excitatory interneurons induce selective, lamina-specific microarchitectural changes in the injured spinal cord. These changes are largely confined to synaptic-level remodeling, including alterations in dendritic spine composition and perineuronal net-associated markers, while global dendritic architecture and dendritic integration remain largely preserved. Despite comparable gross locomotor outcomes between treatment groups, structural analyses reveal distinct patterns of activity-dependent plasticity. This indicates that functional improvement can occur independently of large-scale dendritic or circuit-level reorganization. From a translational perspective, these preliminary findings identify synaptic and perineuronal net-associated microarchitectural substrates as promising but yet to be validated mechanistic targets for refining activity-based neuromodulatory and rehabilitative strategies after SCI, without implying restoration of global neuronal architecture.

## Figures and Tables

**Table 1:** Summary of experimental design and group assignments. ✔ indicates the procedure was performed; - indicates the procedure was not performed. i.t., intrathecal. Group sizes: Sham (n = 8), SCI (n = 10), SCI+TMT (n = 10), BL-OG (n = 10).

Group	Laminectomy vTh8	Contusion SCI	Laminectomy vTh13	AAV9-Hb9-LMO3-EYFP	Treadmill Training	CTZ i.t.
Sham	✔	-	✔	-	-	-
SCI	✔	✔	-	-	-	-
SCI+TMT	✔	✔	-	-	✔	-
BL-OG	✔	✔	✔	✔	✔	✔

**Table 2:** Dendritic spine density in lamina IX of the Th9 and L2 (values are presented as Th9/L2) spinal cord segments. Statistical analysis was performed using the non-parametric Kruskal–Wallis test followed by Dunn’s multiple comparisons post hoc test; *p* < 0.05: * indicates Intact vs. Sham, and ^#^ indicates Sham vs. SCI. Data are presented as mean ± standard error of the mean (SEM).

Spine Type	Intact	Sham	SCI	SCI+TMT	BL-OG
Thin	0.89 ± 0.05 */ 0.91 ± 0.10 *	0.84 ± 0.37 ^#^/ 0.78 ± 0.22 ^#^	2.79 ± 0.13/ 2.64 ± 0.14	2.44 ± 1.05/ 1.32 ± 0.21	3.02 ± 0.09/ 1.10 ± 0.18
Mushroom	0.49 ± 0.03 */ 0.52 ± 0.04	0.66 ± 0.17 ^#^/ 0.59 ± 0.05	1.43 ± 0.58/ 1.07 ± 0.02	2.37 ± 0.79/ 0.76 ± 0.09	0.78 ± 0.11/ 0.64 ± 0.08
Stubby	0.47 ± 0.08/ 0.49 ± 0.08	0.46 ± 0.06/ 0.46 ± 0.06	1.11 ± 0.20/ 1.83 ± 0.09	0.79 ± 0.23/ 1.18 ± 0.15	0.55 ± 0.28/ 0.85 ± 0.14
Wide	0.32 ± 0.07/ 0.33 ± 0.10	0.34 ± 0.09/ 0.20 ± 0.11	0.40 ± 0.006/ 0.36 ± 0.05	0.44 ± 0.65/ 0.31 ± 0.08	0.33 ± 0.17/ 0.28 ± 0.09

## Data Availability

The original contributions presented in this study are included in the article/Supplementary Material. Further inquiries can be directed to the corresponding author.

## Supplementary Materials

The following supporting information can be downloaded at: https://www.mdpi.com/article/10.3390/cells15060571/s1, Supplementary S1: Production of recombinant AAV vectors; Supplementary Figure S1: Tissue sparing analysis at the lesion epicenter; Supplementary Figure S2: Representative Sholl profiles of individual lamina IX motoneurons at the L2 spinal cord level for each experimental group. Supplementary Table S1: Friedman test for BBB locomotor scores.

## Abbreviations

The following abbreviations are used in this manuscript:

SCI	Spinal cord injury
TMT	Treadmill-based motor training
BL-OG	Bioluminescent optogenetic
BBB	Basso, Beattie and Bresnahan
WFA	Wisteria floribunda agglutinin
PNN	Perineuronal net
GPC-4	Glypican-4
vTh8	Vertebrate thoracic segment 8
CTZ	Coelenterazine
Dpi	Days post-injury
PARV	Parvalbumin
