# Supplementary material for: Reorganization of Spinal Cord Microarchitecture by Bioluminescent Optogenetic and Rehabilitative Interventions
Source: Cells. 2026 Mar 23;15(6):571. doi: 10.3390/cells15060571 (PMC13025633; doi:10.3390/cells15060571)
Supplement: Supplementary file 1 [file cells-15-00571-s001.zip › cells-4199007-supplementary.pdf]

## Supplementary S1. Production of Recombinant AAV Vectors

Detailed methods for the production and quality control of recombinant adeno-associated viral (AAV) vectors used in this study are provided below. All procedures were performed as described in Ageeva et al. (2026).

### S1.1. Plasmid Constructs

The plasmid pAAV-Hb9-LMO3 (sbGLuc-VChR1-eYFP) was obtained from Addgene (Watertown, MA, USA; catalog #114472, Addgene plasmid: 114103). Luminopsin 3 (LMO3) is a fusion protein comprising a slow-burn *Gaussia* luciferase variant (sbGLuc), *Volvox* channelrhodopsin-1 (VChR1), and enhanced yellow fluorescent protein (eYFP) under the control of the Homeobox 9 (Hb9) promoter.

### S1.2. Plasmid DNA Amplification

For large-scale plasmid DNA production, the *Escherichia coli* strain NEB® Stable (Thermo Fisher Scientific, Waltham, MA, USA) was transformed with the pAAV-Hb9-LMO3 construct. Competent cells were prepared using the $CaCl_2$ method. Bacterial cells were cultured in low-salt Luria–Bertani (LS-LB) medium (per 1 L of deionized water: salt-free yeast extract, 5 g; tryptone, 10 g; NaCl, 5 g; pH 7.5; all reagents from Sigma-Aldrich, St. Louis, MO, USA) at 37 °C with vigorous shaking (200–300 rpm) until $OD_{600}$ = 0.30–0.35. A single colony of recombinant *E. coli* containing the plasmid was inoculated into 2–5 mL of LS-LB medium with the appropriate selective antibiotic and incubated at 37 °C (300 rpm) for 8 h. The starter culture was then diluted 1:500 in 50 mL of LB medium and incubated at 37 °C (300 rpm) for 12–16 h to reach a cell density of approximately 3–4 × $10^9$ cells/mL. Bacterial cells were harvested by centrifugation at 6000 × *g* for 15 min at 4 °C. Plasmid DNA was isolated by alkaline lysis using the GeneJET Plasmid Miniprep Kit (Thermo Fisher Scientific) according to the manufacturer's protocol.

### S1.3. AAV Production by Triple Transfection

Recombinant AAV9-Hb9-LMO3-EYFP was produced in HEK293T cells (ATCC CRL-11268, American Type Culture Collection, Manassas, VA, USA) by the triple transient transfection method. HEK293T cells were cultured in high-glucose DMEM (Gibco, Waltham, MA, USA) supplemented with 10% (v/v) fetal bovine serum (FBS; Biosera Europe, France) and penicillin–streptomycin at 37 °C in a humidified atmosphere of 5% $CO_2$ and 95% relative humidity. Cell lines were confirmed to be free of mycoplasma contamination by PCR testing. Transfection was performed on a total culture area of 7500 $cm^2$ using the pAAV-Hb9-LMO3 transfer plasmid, an AAV9 capsid plasmid, and an adenoviral helper plasmid.

## S1.4. Purification and Quality Control

Viral preparations were purified from cellular debris, contaminating proteins, and empty viral capsids according to a previously established laboratory protocol [22–24]. The purified vector was sterilized by filtration through a 0.22 µm syringe filter and stored in buffer (1× PBS / 213 mM NaCl / 0.001% Pluronic F-68) in aliquots at −80 °C.

Viral titer was determined by quantitative PCR (qPCR) targeting inverted terminal repeats (ITRs) using the following primer/probe set: forward, 5′-GGAACCCCCCTAGTGATGGAGTT-3′; reverse, 5′-CGGCCTCAGTGAGCGA-3′; probe, 5′-(FAM) CACTCCCTCTCTCTGCGCGCGCTCG (BBQ)-3′. The final titer of the AAV9-Hb9-LMO3-EYFP preparation was $3.52 \times 10^{13}$ GC/mL.

## References

[1] Ageeva, T.; Shigapova, R.; Davletshin, E.; Plotnikova, E.; Rizvanov, A.; Mukhamedshina, Y. AAV9-Mediated Targeting of Defined Neuronal Populations in Spinal Cord Through Intrathecal Injection. *Front. Biosci. (Elite Ed)* **2026**, *18*(1), 44274. https://doi.org/10.31083/FBE44274.

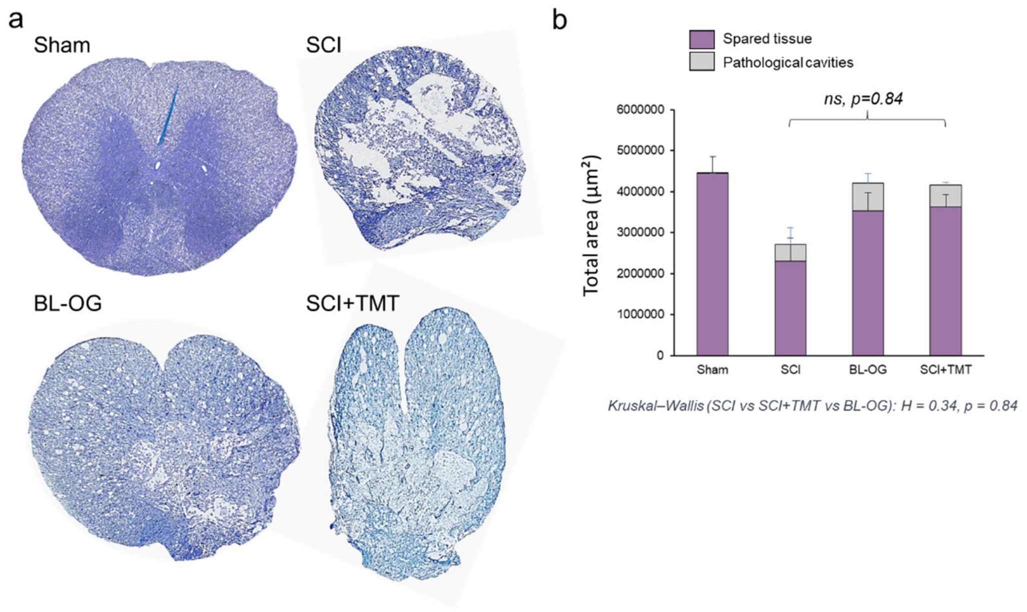


**Supplementary Figure S1.** Tissue sparing analysis at the lesion epicenter. **(a)** Representative azur-eosin–stained transverse sections of the spinal cord at the injury epicenter (0.1 cm rostral to 0.1 cm caudal) in the Sham, SCI, SCI+TMT, and BL-OG groups. **(b)** Stacked bar graph showing the mean area (µm²) of spared tissue and pathological cavities at the lesion epicenter. Error bars represent +SEM. No significant differences in spared tissue were detected among the SCI, SCI+TMT, and BL-OG groups (Kruskal–Wallis, $H = 0.34$, $p = 0.84$).

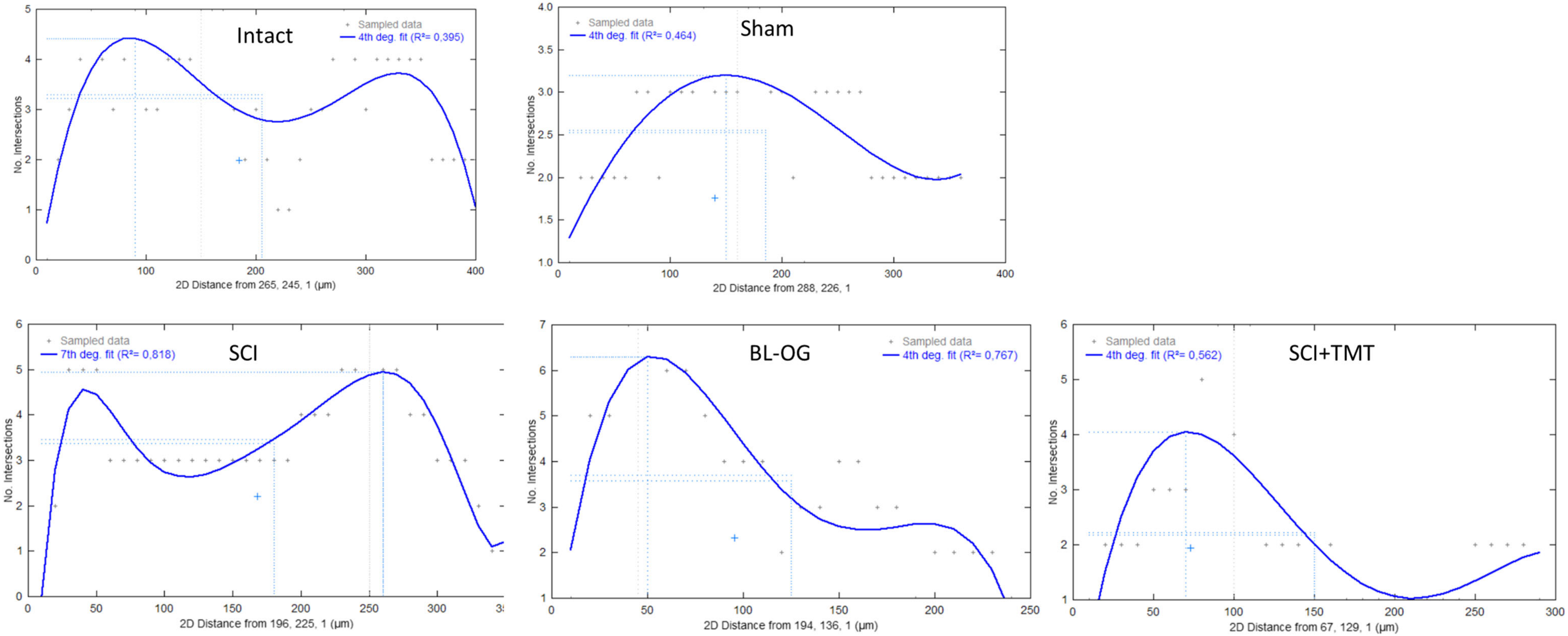


**Supplementary Figure S2.** Representative Sholl profiles of individual lamina IX motoneurons at the L2 spinal cord level for each experimental group. The number of dendritic intersections is plotted as a function of distance from the soma center (step size: 10 µm). Individual profiles correspond to the representative neurons depicted in Figure 4a′. In the Intact and Sham profiles, intersections were distributed across radii up to 350–400 µm, with peak values reaching 4–5 intersections. The SCI profile displayed lower peak intersection counts compared with Intact and Sham, consistent with reduced dendritic branching complexity observed at the group level (Figure 4b). In the BL-OG and SCI+TMT profiles, dendritic arbors were shorter (up to 240–300 µm), with intersections concentrated at proximal radii. These profiles are provided for illustrative purposes; quantitative between-group comparisons are presented in the main text. Polynomial curves were generated automatically by the Fiji Sholl analysis plugin for visualization purposes only.

**Supplementary Table S1.** Friedman test for BBB locomotor scores across time points (7, 14, 18, 21, 25, 28 dpi) within each experimental group. The Friedman test (nonparametric repeated-measures analysis) was used to evaluate whether BBB scores changed significantly over time within each group. n, number of animals with complete data at all time points; df, degrees of freedom. *** $p < 0.001$; ns, not significant.

| Group | Friedman $\chi^2$ | df | p-value | |
|---|---|---|---|---|
| Sham | 8.08 | 5 | 0.152 | ns |
| SCI | 28.03 | 5 | < 0.001 | *** |
| SCI+BL-OG | 28.22 | 5 | < 0.001 | *** |
| SCI+TMT | 39.71 | 5 | < 0.001 | *** |

**Post-hoc analysis** (Wilcoxon signed-rank test, 7 dpi vs. 28 dpi): SCI+TMT, $W = 0.0$, $p = 0.008$; SCI, $W = 0.0$, $p = 0.016$; SCI+BL-OG, $W = 0.0$, $p = 0.031$. All three injured groups showed significant improvement from 7 to 28 dpi, confirming progressive locomotor recovery. The Sham group was not tested post-hoc due to a nonsignificant Friedman result.

*These within-group repeated-measures results complement the between-group Kruskal–Wallis analyses reported in the main text (Figure 2a), confirming that the observed locomotor improvements represent genuine recovery trajectories rather than random time-point variation.*
